# Evaluating male sexual function and reproductive health during Omicron outbreak in China

**DOI:** 10.1371/journal.pone.0310145

**Published:** 2024-11-07

**Authors:** Jiatong Xiao, Bolong Liu, Juliet Matsika, Ronghua Wu, Zheng Tang, Hui Xu, Xiaowei Dai, Guoou Xie, Fabang Liu, Jingeng Dun, Xiongbing Zu, Jinbo Chen, Xiaogen Kuang, Tao Guo

**Affiliations:** 1 Departments of Urology, Xiangya Hospital, Central South University, Changsha, Hunan, China; 2 National Clinical Research Center for Geriatric Disorders, Xiangya Hospital, Changsha, Hunan, China; 3 Department of Andrology, The First Affiliated Hospital, Hengyang Medical School, University of South China, Hengyang, Hunan, China; 4 Department of Urology, Second Affiliated Hospital of Army Medical University, Chongqing, China; 5 Department of Urology, The Affiliated Hospital of Chengde Medical University, Chengde, Hebei, China; 6 Department of Reproductive Medicine Center, The Second Norman Bethune hospital of Jilin University, Changchun, Jilin, China; 7 Department of Urology, Hunan Aerospace Hospital, Changsha, Hunan, China; 8 Department of Urology, The Third Hospital of Mianyang, Sichuan Mental Health Center/The Third Hospital of Mianyang (Sichuan Mental Health Center), Mianyang, Sichuan, China; 9 Department of Urology, Xiangya Changde Hospital, Changde, Hunan, China; 10 Office of Public Health and Medical Emergency Management, Xiangya Hospital, Central South University, Changsha, Hunan, China; 11 Department of Urology, The First Affiliated Hospital, Hengyang Medical School, University of South China, Hengyang, Hunan, China; 12 Learning Alliance of Urology, Changsha, Hunan, China; 13 Department of Urology, The fourth hospital of Changsha, Changsha, Hunan, China; Universite Clermont Auvergne, FRANCE

## Abstract

There are currently no studies exploring omicron infection and male sexual function and semen quality. Our aim was to estimate changes in sexual function and semen quality in men recovering from infection since the COVID-19 Omicron pandemic started in China in late 2022. We collected 1540 questionnaires and assessed male function before infection and acute phase after infection by using International Index of Erectile Function-5, Premature Ejaculation Diagnostic Tool, and Arizona Sexual Experience Scale. We also collected the before and after semen analysis results of 247 male patients. During the acute phase after infection, the proportion of erectile dysfunction was significantly higher than before infection, but ejaculatory function was not significantly altered; In addition, semen analysis showed significant difference in semen concentration, semen activity and PR a+b sperm forward movement after infection compared to pre-infection.: COVID-19 Omicron can affect erectile function as well as sexual experience in male patients in the acute phase. Decreased sexual function due to COVID-19 Omicron may be related to body temperature and anxiety during infection.

## Introduction

In December 2019, a novel coronavirus named SARS-CoV-2 was discovered in Wuhan, Hubei Province, China, and subsequently swept across the country and the world. Since then, countries around the world have adopted different protective quarantine measures to deal with the outbreak [1]. SARS-CoV-2 and SARS-CoV-1 belong to the same coronavirus subfamily, called beta coronaviruses. Over time, a number of variants of SARS-CoV-2 have emerged in the course of transmission, and the Omicron variant is the most variable strain of SARS-CoV-2, and its high transmissibility and immune evasion ability have attracted global attention [2]. Due to its high transmissibility, the Omicron variant has replaced the Delta strain as the dominant strain in many countries worldwide, creating new challenges for the prevention and control of coronavirus disease 2019 (COVID-19). Between March 1 and April 22, 2022, more than 500,000 cases of local infection with omicron were reported in almost all provinces of China [3]. Owing to its population size of over 1.4 billion people, it was inevitable that China would have a large number of patients with symptoms after infection in this round of the epidemic, bringing new challenges to the public healthcare system. During the SARS-CoV-2 and its variants pandemic, researchers worked to investigate the impact of COVID-19 on public health and diseases of various organ systems.

Several studies have previously demonstrated the negative effects of novel Corona infections on men’s sexual health. Sexual health mainly includes sexual function as well as reproductive function health. A research study demonstrated that SARS-CoV-2 infection worsened existing erectile dysfunction(ED) in sexually active male individuals, but the severity of infection was not associated with the occurrence of ED [4]. A study in a European population analyzed the association between COVID-19 genetic susceptibility and ED using Mendelian randomization, and this study showed a causal relationship between COVID-19 genetic susceptibility and increased risk of ED in the European population [5]. A study of a Latin American population also confirmed a negative interaction between the effects of the COVID-19 pandemic and erectile/sexual function [6]. The results of a meta-analysis showed that restriction associated with COVID-19 was associated with higher rates of sexual dysfunction and reduced sexual activity, suggesting that the infection adversely affected both the quantity and quality of sexual activity [7]. In addition to the investigation of the direct effects on sexual function, there are also a number of studies that have underscored the role of poor mental health issues in this regard [8]. Many studies have also confirmed that the level of depression and the level of sexual dysfunction are directly correlated [9].

In addition to sexual dysfunction, reproductive health has also received some degree of influence from COVID-19 infections, as confirmed by some basic experimental studies and clinical research. A study confirms high expression of SARS-CoV-2 receptor angiotensin-converting enzyme 2 (ACE2) in human testis and spermatozoa [10], It also provides evidence for the expression of co-receptor transmembrane protease/serine (TMPRSS2), Basigin (BSG) and CatepsinL (CTSL). In addition, bioinformatics tools suggest the same view, as disruption of spermatogenesis and reduced expression of spermatogenesis-related genes were observed [11]. A multi-organ proteomic study of COVID-19 autopsies showed testicular damage after infection, with evidence of damage including reduced testicular interstitial cells, inhibition of cholesterol biosynthesis, and sperm activity [12]. The above studies reveal the negative effects of COVID-19 infection on male sexual function and semen quality, but the effects of the Omicron variant on male sexual function and semen quality in mainland China are not clear.

As a result of changes in China’s anti-epidemic policy, China has seen a large number of patients with Omicron infections, and many patients present to urology with concerns about their sexual function and reproductive health. To have a better understanding of this problem, we used an online questionnaire combined with the analysis of semen samples, aiming to compare the sexual function and semen sperm quality of men infected with omicron over different time spans, to explore the impact on the sexual health of infected men in the context of this pandemic, and to provide some reference value for the treatment of male patients after becoming infected.

## Method

### Study design and participants

A cross-sectional study was designed to investigate the effect of an Omicron infection on the sexual function and semen quality of men. The study was approved by the Ethics Committee of Xiangya Medical College (202305361). After ethical approval was obtained, we designed and distributed an online questionnaire containing an electronic informed consent form, informed consent was provided at the beginning of the questionnaire, the completion of which represented the informed consent of the subject. We also collected semen analysis results before and after infection with Omicron from some of the male patients attending the hospital offline, so all participating patients signed an informed consent form. The subjects of this study are recovered male patients infected with Omicron. Male recovered patients with Omicron who meet the following inclusion or exclusion criteria will be included or excluded from this study.

Inclusion criteria: (1) male patients between 18 and 60 years of age; (2) male patients with Omicron infection diagnosed by positive nucleic acid or antigen test; (3) consent and signed informed consent; (4) having had six months of regular sexual intercourse before contracting SARS-CoV-2. (5) semen analysis group inclusion criteria: semen analysis was performed within 2 months before infection.

Exclusion criteria: (1) history of genitourinary or pelvic trauma or surgery; (2) severe cardiovascular disease; (3) uncontrolled hypertension or diabetes mellitus, or other serious chronic diseases; (4) history of alcohol or narcotic drug abuse, drug use, or a history of psychiatric disorders (e.g., schizophrenia, obsessive-compulsive disorder, depression), antagonistic personality, poor motivation, paranoia, or other emotional or intellectual problems that may affect the informed validity of participation in this study; (5) patients who are unable to cooperate with the tests associated with this project and do not agree to sign the informed consent form.

### Procedure

The study was conducted after gradual easing of restrictions by the Chinese government, and a questionnaire was designed and distributed in early 2023. A total of 1540 questionnaires were collected from 03/05/2023 to 16/06/2023, and the questionnaires were screened for inclusion and exclusion based on the questionnaire questions, resulting in 1363 valid questionnaires. Meanwhile, after some patients signed an informed consent form, we conducted a collection of semen analysis results from 01/05/2023 to 16/06/2023 for before and after infection in our offline male clinic, and obtained semen analysis results from a total of 247patients. The participants’ identifiable information was blurred to ensure that their personal information would not be identified.

### Measure

Erectile function was assessed according to the validated Chinese version of the IIEF-5 questionnaire, with 5 scales from 1 to 5 assessing maintenance ability, confidence in erection, frequency of maintenance, erectile hardness, and satisfaction with intercourse. A total score of 22–25, 12–21, 8–11 and 5–7 was considered to represent normal, mild, moderate and severe ED, respectively. the threshold for ED was designated as 21 [13].

Premature ejaculation was assessed according to the Chinese version of the premature ejaculation diagnostic tool (PEDT). The scale is assessed by a combination of 5 basic questions. A total score ≥ 11 indicates the presence of premature ejaculation problems (ejaculatory control dysfunction); a total score between 9 and 10 indicates the possible presence of premature ejaculation problems; if the total score ≤ 8, it indicates the absence of premature ejaculation problems [14].

The Arizona Sexual Experience Scale (ASEX) is a self-report inventory containing five items. It measures sexual function in both men and women regardless of their sexual orientation or relationship with their partner. It measures the quality of sexual function through five questions, each representing one domain: sexual desire, sexual arousal, penile erection/vaginal lubrication, ability to achieve orgasm, and satisfaction from orgasm. The scores for each item were aggregated. Clinical sexual dysfunction was identified if a total score of >19 was observed, and/or a score of >5 for any one item, and/or a score of >4 for any three items. The Cronbach α value for ASEX in the study sample was 0.83 [15].

### Ethics approval and consent to participate

The study was approved by the Ethics Committee of Xiangya Medical College (202305361). we designed and distributed an online questionnaire containing an electronic informed consent form, informed consent was provided at the beginning of the questionnaire, the completion of which represented the informed consent of the subject. We also collected semen analysis results before and after infection with Omicron from some of the male patients attending the hospital offline, so all participating patients signed an informed consent form.

### Statistical analysis

All questionnaire data were stored electronically in SPSS statistical software version 25 (IBM) for statistical analysis. Pearson’s chi-square test or Fisher’s exact test was used to assess the statistical significance of categorical variables. Independent samples t-tests and multivariate logistic regression analyses were performed to explore risk factors. For the results of semen analysis, we used a nonparametric single sample t test. Statistical significance was considered to be P < 0.05.

## Results

In this research, 1540 questionnaires were collected from May 3, 2022 to June 16, 2023, and questionnaires were screened for inclusion and exclusion based on the questionnaire questions, detailed information in S1 Table. The final number of valid questionnaires was 1363. We used scales to assess male function and semen quality in the target population before infection and the acute phase after infection, detailed information in S2 Table. We also paid attention to the influence of psychological factors. After patients signed an informed consent form, we conducted a collection of semen analysis results before and after infection in an offline male clinic, and obtained semen analysis results from a total of 247 patients. Statistical methods were used to analyze the above questions. The baseline demographic and clinical characteristics of the patients are shown in Table 1. where the median age of the subjects was at 38 years and the median BMI was 23.88; 59.3% explicitly stated that they were concerned about sexual function and 52% stated that they were concerned about sexual function.

**Table 1:** Demographics, clinical characteristics of Omicron recovered patients.

Characteristic	Number	Frequency(%)
**Age[38(18–81)years]**		
18-35years	568	41.7
36-50years	691	50.7
51-65years	95	6.9
≥66years	9	0.7
**Height[171(142–196)cm]**		
140-155cm	13	1.0
156-170cm	669	49.0
171-185cm	662	48.6
≥186cm	19	1.4
**Weight[70(44–110)kg]**		
40-55kg	95	7.0
56-70kg	661	48.5
71-85kg	503	36.9
≥86kg	104	7.6
**BMI[23.9(14.7–34.7)kg/m** ^**2**^ **]**		
14-20kg/m^2^	123	9.0
21-25kg/m^2^	774	56.8
26-30kg/m^2^	421	30.9
≥30kg/m^2^	45	3.3
**Education**		
Primary education	17	1.2
Middle education	185	13.6
Specialty	254	18.6
Undergraduate college	600	44.0
Postgraduate	307	22.5
**Smoke**		
Yes	531	39.0
No	832	61.0
**Drink**		
Yes	591	43.4
No	772	56.6
**Vaccination**		
unvaccinated	30	2.2
Get a one-dose vaccine	30	2.2
Get a two-dose vaccine	209	15.3
Get the three-dose vaccine	1005	73.7
Get the four-dose vaccine	89	6.5
**Had urinary problems in the past 12 months**		
Yes	306	22.5
No	1057	77.5
**Body temperature during infection**		
No fever	68	5
37.1–38°C	391	28.7
38.1–39°C	593	43.5
39.1–40°C	276	20.2
40.1–41°C	29	2.1
>41°C	6	0.4
**Hospitalization during infection**		
Yes	1302	95.5
No	61	4.5
**Worried about sexual function**		
Don’t worry	437	32.1
Don’t know	116	8.5
A little	591	43.4
Worried very much	219	16.1
**Worried about reproductive function**		
Don’t worry	536	39.3
Don’t know	118	8.7
A little	512	37.6
Worried very much	197	14.5

BMI: Body Mass Index.

Table 2 show the scores on the three scales before and after infection with omicron and the frequency table of the degree of prevalence. 656 subjects (51.9%) showed mild to severe erectile dysfunction before infection, and this value changed to 76.3% after infection. Premature ejaculation or suspected patients accounted for 30.4% and 32.6% before and after the infection, respectively; while patients with sexual dysfunction according to the ASEX scale accounted for 15.4% and 45.9%, respectively.

**Table 2:** Frequency distribution of three scales before and after Omicron infection.

	Before Infection		After Infection		P value
	N	%	N	%	
**IIEF-5**					<0.05
No erectile dysfunction (>21)	656	48.1	323	23.7	
Mild erectile dysfunction (12–21)	418	30.7	374	27.4	
Moderate erectile dysfunction (8–11)	68	5.0	118	8.7	
Severe erectile dysfunction (<8)	221	16.2	548	40.2	
**PEDT**					0.489
No premature ejaculation (≤8)	948	69.6	919	67.4	
Suspected premature ejaculation (9/10)	153	11.2	163	12	
Premature ejaculation(≥11)	262	19.2	281	20.6	
**ASEX**					<0.05
No sexual dysfunction	1153	84.6	738	54.1	
Sexual dysfunction*	210	15.4	625	45.9	

IIEF-5: International Index of Erectile Function-5; PEDT: Premature Ejaculation Diagnostic Tool; ASEX: Arizona Sexual Experience Scale.

In addition, we performed Pearson chi-square statistical difference analysis of the scores of the three scales before and after infection with Omicron (Table 3), and we found that the erectile function scale IEEF-5 and the sexual experience scale ASEX scores were significantly different before and after infection with Omicron (p < 0.05), while the premature ejaculation function scale PEDT scores did not differ before and after infection with omicron (p = 0.489).

**Table 3:** Analysis for the risk factors of erectile dysfunction.

	Univariate analysis	Multivariate analysis		
	P	OR	95% CI	P
**Age**	<0.001	1.036	1.023–1.049	<0.001
**Height**	0.011	0.996	0.977–1.016	0.708
**Weight**	0.21			
**BMI**	0.154			
**Education**	<0.001	1.221	1.076–1.386	0.002
**Smoke**	<0.001	0.689	0.541–0.878	0.003
**Drink**	0.474			
**Vaccination**	<0.001	1.086	0.912–1.293	0.353
**Had urinary problems in the past 12 months**	0.016	1.04	0.784–1.381	0.784
**Body temperature during infection**	<0.001	1.253	1.096–1.434	0.001
**Hospitalization during infection**	<0.001	0.653	0.463–0.921	0.015
**Worried about sexual function**	<0.001	1.590	1.371–1.843	<0.001
**Worried about reproductive function**	0.754			

BMI: Body Mass Index; OR: odds ratio

The scale scores of 1363 patients before and after infection with Omicron were subtracted to divide them into "worse" and "not worse" components, and then independent sample t-tests and multivariate logistic regression were used to analyze the possible risk factors (Table 4). Factors with P values less than 0.05 in univariate analysis were included in the multivariate model. It can be seen that in the results of multivariate analysis, "education", "smoking or not", "temperature during infection", "hospitalization during infection or not ", "concern about sexual function" and "age" may be related to ED (p < 0.05). While "education", "whether or not you have had urinary problems in the past 12 months", "body temperature during infection" and "whether or not you are worried about sexual function " were related to sexual function (p < 0.05).

**Table 4:** Analysis for the risk factors of sexual experience.

	Univariate analysis	Multivariate analysis		
	P	OR	95% CI	P
**Age**	0.255			
**Height**	0.085			
**Weight**	0.849			
**BMI**	0.877			
**Education**	<0.001	1.206	1.055–1.377	0.006
**Smoke**	0.017	0.954	0.741–1.228	0.714
**Drink**	0.07			
**Vaccination**	0.213			
**Had urinary problems in the past 12 months**	<0.001	1.558	1.172–2.071	0.002
**Body temperature during infection**	0.048	1.164	1.013–1.338	0.032
**Hospitalization during infection**	0.488			
**Worried about sexual function**	<0.001	1.549	1.166–2.058	0.003
**Worried about reproductive function**	0.419			

OR: odds ratio

The results of semen analysis mainly included semen volume (ml), sperm concentration (million /ml), total sperm count (million), total motility (%), PR a+b sperm forward movement (%) and normal sperm morphology (%). Due to the small sample size, we conducted K-S test and found that the samples did not conform to the normal distribution. Therefore, a non-parametric independent sample T-test was conducted on all data before and after infection (Table 5), and significant differences were found in semen concentration, semen activity and PR a+b sperm forward movement before and after infection (p< 0.05).

**Table 5:** Non-parametric independent sample T-test of semen analysis before and after Omicron infection.

	Volume	Concentration	Activity	Number	PR	Form
**Mann-Whitney U**	26288	25760.5	24913	18684.5	24721	1311.5
**Wilcoxon W**	56423	56141.5	55048	40420.5	55102	2439.5
**Z Scores**	-1.723	-2.211	-2.623	-1.696	-2.889	-0.031
**P**	0.085	0.027	0.009	0.09	0.004	0.975

Volume: semen volume (ml), Concentration: sperm concentration (million /ml), Activity: total motility (%), Number: total sperm count (million), PR: PR a+b sperm forward movement (%), Form: normal sperm morphology (%)

## Discussion

Increased globalization and more frequent interactions led to COVID-19 sweeping through the globe in a few months. The introduction of countermeasures in different countries and the generational changes the virus has undergone over the past three years allowed China to gradually loosen its COVID-19 precautions on December 26, 2022, after a comprehensive assessment of the possible consequences. There was a steady increase of Omicron infection amongst the Chinese population from this point on and the symptoms associated with COVID-19 were not only limited to the respiratory system, but also to the digestive [16] and nervous systems [17]. Many male patients have reported a link between this omicron infection and decreased male reproductive function, including sexual health and semen quality. To answer these questions, we explored the relationship between Omicron and erectile function, ejaculatory function and sexual experience in men from different perspectives, and we also collected the results of semen analysis before and after Omicron infection in some patients in an attempt to explore the effects of Omicron infection on male semen quality.

ED is the inability to maintain or achieve an erection sufficient for satisfactory sexual intercourse. To further investigate the effect of omicron infection on erectile function in men, we calculated the IEEF-5 scores of each patient before and after Omicron infection and categorized them as 21–30 (no ED), 12–21 (mild ED), 8–11 (moderate ED) and <7 (severe ED), according to the scale. Statistical analysis was performed, and the results proved that there was a significant difference in erectile function in men before and after infection with Omicron, and that infection with Omicron contributed to the development of erectile dysfunction to some extent. Several studies have been conducted to discuss the correlation between COVID-19 infections and male erectile function. SARS-CoV-2 viral infections not only causes respiratory damage, but may also have serious effects on the male patients’ reproductive system which regulate many physiological processes. Even though it appears that erectile function deteriorates after COVID-19 infection, the function tends to improve over time [18] Excessive inflammation and immune suppression are prominent in COVID-19, leading to cytokine storms, ultimately resulting in the development of microthrombosis and disseminated intravascular coagulation (DIC). PDE-5 inhibitors approved for the treatment of erectile dysfunction have anti-inflammatory and antioxidant effects, and are also being studied for the treatment of COVID-19 patients [19]. Some researchers have also characterized the histopathology of tissue from patients who recovered from symptomatic COVID-19 infection, finding that the COVID-19 virus is present in the penis long after the initial infection in human [20]. Interestingly, an article suggested that PDE5 inhibitors, therapeutic agents for erectile function, may target potential targets of COVID-19 due to their anti-inflammatory, antioxidant, immune response modulating and anti-apoptotic properties [21]. Meanwhile, a cross-sectional study showed that COVID-19 vaccination did not affect male sexual function, including erectile function [22]. However, no studies were found that examined the effects of infection with Omicron on male erectile function.

In order to investigate the risk factors for ED due to Omicron infection, we conducted multivariate logistic regression analysis and finally found that "education", "smoking or not", "body temperature during infection", "hospitalization or not during infection", "concern about sexual function" and "age" were possible related to ED. We speculate that there may be differences in the acquisition of knowledge about prevention, so that education becomes a related factor, while smoking history, temperature, and hospitalization or not may respond to the severity of symptoms from different aspects, especially endothelial cell disorders due to severe inflammation as indicated by high temperature, which has been studied and proven to be a possible factor contributing to ED.

Since male sexual function includes not only erectile function but also ejaculatory function and sexual experience. There are very few studies on the effects of COVID-19 infection on premature ejaculation and sexual experience, and no articles have been found on the relationship with Omicron either. As premature ejaculation is an important male disease, it is necessary to explore its relationship with Omicron, so we found that 11.4% of men had premature ejaculation or worsened premature ejaculation after infection with Omicron based on the scores of PDET before and after infection with Omicron obtained from the questionnaire, but the results of the difference analysis revealed that the incidence of premature ejaculation before and after infection was not statistically significant. The same approach was used to analyze the scores of the ASEX scale and it was found that 31.5% of the patients experienced a decrease in sexual experience after infection with Omicron, with statistically significant differences before and after infection. Multivariate logistic regression was used to assess related factors, and it was found that "education", "whether or not you had urinary tract problems in the past 12 months", "body temperature during infection "and "concern about sexual function" were found to be related factors for decreased sexual experience due to Omicron infection. A number of studies have examined the impact of anxiety during the COVID-19 epidemic on the occurrence of sexual dysfunction. During the COVID-19 epidemic, a certain percentage of adult men were at increased risk for premature sexual ejaculation. The postponed fertility treatment brought about by the COVID19 epidemic has led to an increase in the proportion of patients with premature ejaculation, probably due to anxiety [23]. A study of the complications of COVID-19 infection in patients with epilepsy noted that outbreaks of COVID-19 led to an increased propensity for depression in patients with epilepsy and also negatively impacted sexual experience [24].

Many researchers have speculated that in addition to being present in the respiratory system and transmitted by droplets, SARS-COV-2 virus may also be present in the reproductive system and transmitted by body fluids, and many studies have emerged to test this speculation. Investigation of the molecular details of SARS-CoV-2 infection has been rapidly initiated, and several key facts are already known. Viral entry requires SARS-CoV-2 spike-in(S) glycoprotein to bind to host ACE2. Host TMPRSS2 is then required to cleave the viral S protein to induce a conformational change that allows permanent fusion of the viral and host cell membranes [25]. The results of single-cell sequencing confirmed that co-expression of ACE2 and TMPRSS2 was not detected in testicular cells (including spermatozoa) [26]. A prospective longitudinal cohort provided direct experimental evidence that the male reproductive system may be targeted and disrupted by COVID-19 infection by assessing semen ACE84 activity, inflammatory and oxidative stress markers, apoptotic variables, and semen quality parameters [27].

On the basis of questionnaire surveys to explore male sexual function, we collected the semen analysis results of 247 patients before and after infection in the andrology clinic of the hospital, mainly from the semen volume, sperm concentration, total number of sperm, PR a+b sperm forward movement, sperm survival rate and proportion of normal sperm are used to comprehensively evaluate the patient’s semen quality. Based on the 247 semen analysis results obtained, we found the impact of COVID-19 infection on semen analysis data including semen concentration, semen activity and PR a+b sperm forward movement, which is consistent with the results of some previous basic research. An autopsy of semen specimens from China for sperm parameters and immune factors also revealed impaired spermatogenesis and the development of autoimmune orchitis, presumably due to elevated levels of local immune responses [12]. Many analyses of sperm parameters in semen samples have shown a significant correlation between COVID-19 infection and decreased sperm quality [28]. Additional analyses of oxidative stress markers and sperm DNA for semen samples suggest that increased DNA fragmentation and decreased semen quality in men may be the result of an imbalance between semen precursors and antioxidant components after COVID-19 [29]. The research results from electron microscopy indicate that sperm production is based on nuclear DNA extracellular traps, which may be generated in a DNA-independent manner, similar to the traps described previously in the systemic inflammatory response to COVID-19 [30].

In this study, we investigated for the first time the effects of Omicron on sexual function and semen quality in Chinese men by collecting male sexual function questionnaires from men infected with Omicron and analyzing the semen of some patients, and also found adverse effects of Omicron infection on male erectile function, male sexual experience and male semen quality. Anxiety and worry were involved as confounding factors, while academic qualifications, body temperature during infection, and anxiety during infection were related factors for changes in semen quality with Omicron. Of course, our study has some drawbacks: It is an observational study, which limits the inference of causes; The distribution of the subjects collected is not evenly scattered throughout the provinces of China, but may be concentrated in certain provinces; Due to the small sample size of semen analysis, the correlation between COVID-19 infection and semen quality of patients was not found; The study is limited by the number of questions and does not explore in more detail the effects of various emotional and psychiatric disorders on the process; The collection of data on the scale before and two weeks after the novel coronavirus infection does not allow the analysis of changes in male sexual function and semen quality after a long period of Omicron infection. Pre-infection and acute phase data were collected simultaneously during the acute phase and there may be memory bias. The investigation of sexual function should involve the selection of participants to ensure that they had sexual activity before being infected with SARS-CoV-2. This non-prospective study may have sample selection bias in the inclusion of subjects due to the main research locations being outpatient and inpatient settings. We used a targeted before-after comparison method to analyze the differences in changes before and after infection.

## Conclusion

Since the surge of Omicron pandemic in China from December 2022, male erectile function, sexual experience is negatively affected to some extent. Academic qualifications, body temperature during infection, and anxiety during infection may be related to decreased sexual function due to Omicron. At the same time, we find a correlation between COVID-19 infection and semen concentration, semen activity and PR a+b sperm forward movement in patients.

## Supporting information

S1 Table The original data of survey. (XLSX)

S2 Table Semen analysis raw data. (XLSX)
